# Supplementary material for: Arctic zircon U-Pb ages reveal multiphase glaciations in East Siberia during the late Quaternary
Source: Nat Commun. 2025 Aug 13;16:7511. doi: 10.1038/s41467-025-62499-y (PMC12350766; doi:10.1038/s41467-025-62499-y)
Supplement: Supplementary file 1 — Supplementary Information [file 41467_2025_62499_MOESM1_ESM.pdf]

Supplementary information for

Arctic zircon U-Pb ages reveal multiphase glaciations in East Siberia during the late Quaternary

Han Feng†, Zhengquan Yao†, Xuefa Shi*, Zhongshi Zhang*, Huayu Lu, Hanzhi Zhang, Yanguang Liu, Xin Shan, Jiang Dong, Linsen Dong, Gongxu Yang, Limin Hu, Yuri Vasilenko, Anatolii Astakhov, Alexander Bosin

†These authors contributed equally to this work: Han Feng, Zhengquan Yao.

*Corresponding authors: Xuefa Shi and Zhongshi Zhang

Email: xfshi@fio.org.cn; zhongshi.zhang@pku.edu.cn.

The PDF file includes:

- Supplementary Text S1 to S5
- Supplementary Figures S1 to S14
- Supplementary Tables S1 to S2
- SI References

Other supporting materials for this manuscript include the following:

- Supplementary data S1 to S4

SUPPLEMENTARY TEXT

S1. Challenges in distinguishing the East Siberian source

Here we present previously used provenance tools in Arctic IRD and discuss their challenges in distinguishing the East Siberia source among circum-Arctic continental shelves.

Sr-Nd isotope composition. Complied Sr-Nd isotopic values of surface sediments on the circum-Arctic continental shelves[1,2] show that Sr-Nd isotope composition can effectively distinguish sources between North American continent and the entire Eurasian continent, but have difficulties in distinguishing sources between eastern (East Siberia) and western-central Eurasian continents (Supplementary Fig. S1). On the North American continental shelf, surface sediments show relatively low ε_{Nd} values (mostly < -12) and high $^{87}Sr/^{86}Sr$ values (mostly > 0.720). On the entire Eurasian continental shelf (including East Siberia, central Eurasia, and western Eurasia), on the contrary, surface sediments show relatively high ε_{Nd} (mostly -14 to -6) and low $^{87}Sr/^{86}Sr$ (mostly 0.710 to 0.720). This obvious difference makes Sr-Nd isotope composition effective to differentiate sources between the North America and the entire Eurasia. However, within the Eurasian continental shelf, the range of Sr-Nd isotope values were overlapped between different part of the shelf (Supplementary Fig. S1). On the East Siberian shelf (including the Chukchi Sea and the East Siberian Sea) that was proposed to be covered by the East Siberian Ice Sheet during some glacial periods, surface sediments show ε_{Nd} values mostly ranging from -12 to -7 and $^{87}Sr/^{86}Sr$ mostly ranging from 0.710 to 0.720. On the central Eurasian shelf (Laptev Sea), surface sediments show ε_{Nd} values mostly ranging from -14 to -8 and $^{87}Sr/^{86}Sr$ mostly ranging from 0.712 to 0.720. On the western Eurasian shelf (including the Kara Sea and the Barent Sea) that was proposed to be covered by the Eurasian Ice Sheet during some glacial periods, surface sediments

show ε_{Nd} values mostly ranging from -14 to -7 and $^{87}Sr/^{86}Sr$ mostly ranging from 0.710 to 0.720. These overlapped Sr-Nd isotope values makes Sr-Nd isotope composition ineffective to differentiate sources within the entire Eurasian continental shelf. Therefore, if relatively high ε_{Nd} and low $^{87}Sr/^{86}Sr$ values were identified in Arctic sediments, their source could be either East Siberia or the western-central Eurasia.

Heavy mineral assemblage. The distribution of heavy minerals in surface sediments on circum-Arctic continental shelves (Supplementary Fig. S2)[3] shows that no heavy mineral type can distinctly represents the East Siberian continental shelf. Amphiboles are abundant in the Laptev Sea, eastern East Siberian Sea and southern Barents Sea; Clinopyroxenes have high proportions in western Laptev Sea, southern Kara Sea and northern Barents Sea; Epidotes are accumulated in the Barents Sea, southern Kara Sea, and eastern Chukchi Sea. Garnets have high content in the Barents Sea. However, although the East Siberian continental shelf (including East Siberian Sea and Chukchi Sea) has high amphibole content in its western part and high epidote content in its eastern part, these two heavy mineral types—which are also abundant in other shelf—cannot fingerprint the East Siberian continental shelf.

Quartz and Feldspar content. Quartz and feldspar content and ratios are ineffective in distinguishing East Siberian shelf sediments from other shelves[4]. Alkali feldspar is highest in the Laptev Sea (~20%) and present at ~10–15% in other shelves. Quartz is abundant (~60%) in the North American continental shelf, western Kara Sea, western Laptev Sea, and eastern Chukchi Sea, but lower (~40%) in the eastern Laptev and East Siberian Seas. Plagioclase feldspar peaks at Kara Sea river mouths (~40%) and is ~30% in the western Laptev and East Siberian Seas. The quartz-to-feldspar ratio is highest in the North American shelf and Kara Sea (~3) but lower in the

East Siberian Sea (~1). The plagioclase-to-alkali feldspar ratio is higher in the North American continental shelf but lower in Eurasian shelves (<1). In summary, no specific quartz or feldspar parameter reliably identifies East Siberian sources.

Fe oxide geochemistry. One of the earliest and successful attempts at provenance analysis of Arctic ice-rafted debris (IRD) is documented in a series of studies[5-7]. These studies utilized Fe oxide geochemistry to differentiate between circum-Arctic sediment sources. They collected extensive surface samples from continental shelves across the Arctic and extracted Fe oxide grains, which were then analyzed for 14 elements using an electron microprobe. To establish sample-to-source correlations, they compared the elemental composition of Fe grains from core samples with those from potential source regions, applying a two standard deviation threshold based on replicate analyses to ensure accuracy[6]. They suggest that the average error of mismatches in each source area was 2% or less[6]. Although this provenance tool has been effective in tracing IRD in the Arctic[5-11], the contribution from East Siberia has not been effectively detected in these studies. For example, in core samples from the central Arctic and Fram Strait, fewer than five Fe grains (out of 100 grains analyzed) were matched to East Siberia as the source[9,10]. This suggests that East Siberia's contribution is likely less than 2% (5/100), with its signal possibly being masked by the method's mismatch error (2%)[6].

S2. Provenance of detrital zircon age populations.

The detrital zircon U-Pb ages in both surface and core samples analyzed in this study show a close correlation with magmatic rocks in the respective source regions (Fig. 1). Approximately 95% of the zircons in our samples have Th/U ratios greater than 0.1 (Fig. S14), indicating a

predominantly magmatic origin[12]. While recycled zircons—magmatic zircons temporarily stored in continental sedimentary rocks and subsequently re-eroded and transported to the shelf—can inevitably influence the detrital zircon U-Pb age distribution, they do not obscure the magmatic signal[13]. This is because the ultimate origin of recycled zircons remains the magmatic rocks in the source area. Therefore, in the following discussion of zircon age provenance, we focus exclusively on the magmatic origin of the zircon populations.

We present the potential sources of detrital zircon U-Pb age populations in surface sediments from circum-Arctic continental shelf (Fig. 2), except of the age populations of ~90-110 Ma and ~60 Ma that have been mentioned in the main text. In the Barents Sea, a small age peak at ~185 Ma appears in the surface sediment near the Pechora River Mouth (Fig. 2A), probably reflecting a source from the Ural Mountain formed in the continental collision during the Early Jurassic[14]. In the Kara Sea, two age peaks at ~140-160 Ma and ~180-200 Ma (Figs. 2B-F) indicate the source from the Central Asian Orogenic Belt, located in the upper reaches of the Yenisei and Ob rivers[15]. In the Laptev Sea, the peak at ~140-160 Ma (without the peak at 180–200 Ma) (Figs. 2G-K) is likely sourced from the Kolyma-Omolon Magmatic Belt (~158–129 Ma) (Fig. 1)[16]. In addition, surface sediments from Eurasian continental shelf share prominent zircon age peaks at ~220–360 Ma, ~420–560 Ma, ~1750–2000 Ma, and ~2500–3000 Ma (Fig. 2A-U). The late Carboniferous to Permian (such as peaks at 250 Ma, 286 Ma, and 300 Ma) zircons are likely sourced from the Siberian Traps (Fig. 1)[17] or the Central Asian Orogenic Belt[18] that drained by upper reaches of the Lena, Yenisei, and Ob rivers. Zircons dating back to the Cambrian-Silurian era (~560-420 Ma) are also produced in the northern margin of the Central Asian Orogenic Belt[19]. The most ancient ~2500-3000 Ma zircons that represent the Siberian Archean cratons, as well as the ~1800-2000 Ma zircons that signify the formation of the Siberian craton during the assembly of the Early

Proterozoic Columbia supercontinent[20]. On the North American continent, surface sediments from the Mackenzie River Mouth lack zircon grains with ages <1000 Ma, instead contain numerous zircons within the ranges of ~1000–1500 Ma and ~1800–2000 Ma (Fig. 2W)[21], which are associated with the North American cratons (Fig. 1).

S3. Theoretical Calculations of Particle Initiation and Settling

In the main text, we demonstrate that sea ice rarely contains sediment particles larger than 250 μm, as the shelf regions where sea ice entrains sediments generally lack such coarse particles (Fig. 5). We also demonstrate that zircon grains larger than 150 μm are scarce in this shelf area (Fig. 6), likely due to the higher density of zircon, which makes it harder to transport by rivers and current compared to lighter minerals like quartz and feldspar. Using incipient motion theory and settling theory of the sediment particle, we will estimate the equivalent zircon grain size corresponding to a 250 μm quartz grain under identical hydrodynamic conditions on the continent and continental shelf.

Incipient motion theory. The flow strength at which sediment movement first begins (i.e. incipient motion) is usually expressed in terms of a critical shear stress[22,23]:

$$\tau_c = \tau_c^* g(\rho_s - \rho_w)D \tag{1}$$

Where ρ_s is the particle density, ρ_w is the fluid density, D is the particle diameter, g is the gravitational acceleration, and τ_c^* is the Shields parameter. The Shields parameter is related to the Reynolds number, but their relationship is typically empirical, with values generally ranging between 0.3 and 0.6[24]. Although particle shape influences the Shields parameter[25], it does not alter

this range. Additionally, it is suggested that particle density has little effect on Shields parameter[26,27]. Therefore, under identical hydrodynamic conditions, we can reasonably assume that the Shields parameter for zircon and quartz is equal.

Assuming that the critical shear stress is the same for both quartz (τ_{cq}) and zircon (τ_{cz}) grains, we have:

$$\tau_{cq} = \tau_{cz} \tag{2}$$

From equation (1), this gives:

$$\tau_c^* g(\rho_{sq} - \rho_w)D_q = \tau_c^* g(\rho_{sz} - \rho_w)D_z \tag{3}$$

Where ρ_{sq} (2.648 g/cm³) and ρ_{sz} (4.669 g/cm³) are the densities of quartz and zircon grains, respectively[28]. Then we obtain that, when the diameter of quartz (D_q) is 250 μm, the corresponding zircon grain size (D_z) is 113 μm. In other words, the hydrodynamic conditions capable of initiating the movement of a 250 μm quartz grain can only initiate the movement of a 113 μm zircon grain.

Settling theory. The formula to estimate the settling velocity of non-cohesive particles was first proposed by Ref. 29 and later modified by subsequent studies[30]. Here, we use the formula introduced by Ref. 31 which takes particle shape into account:

$$w_s = \frac{M\nu}{ND}\left[\sqrt{\frac{1}{4} + \left(\frac{4N}{3M^2}d_*^3\right)^{\frac{1}{n}}} - \frac{1}{2}\right]^n \tag{4}$$

Where w_s is the settling velocity of the particle, ν is the kinematic viscosity (for water, the value is 1.003×10^{-6} m²/s at 20°C), and D is the grain size. *M, N,* and *n* are coefficients related to the particle shape:

$$M = 53.5e^{-0.65S_F} \tag{5}$$

$$N = 5.65e^{-2.5S_F} \tag{6}$$

$$n = 0.7 + 0.9S_F \tag{7}$$

S_F is the particle shape factor, with a range of 0.3 to 1.0. For natural particles, a typical value is 0.7[32]. The d_* in equation (2) is the nondimensional grain size:

$$d_* = D\left(\frac{Rg}{\nu^2}\right)^{\frac{1}{3}} \tag{8}$$

Where, $R = \rho_s/\rho_w - 1$.

Assuming that the settling velocity is the same for both quartz (w_{sq}) and zircon (w_{sz}) grains, we have:

$$w_{sq} = w_{sz} \tag{9}$$

From equation (4), this gives:

$$\frac{M\nu}{ND_q}\left[\sqrt{\frac{1}{4}+\left(\frac{4N}{3M^2}d_{*q}^3\right)^{\frac{1}{n}}}-\frac{1}{2}\right]^n = \frac{M\nu}{ND_z}\left[\sqrt{\frac{1}{4}+\left(\frac{4N}{3M^2}d_{*z}^3\right)^{\frac{1}{n}}}-\frac{1}{2}\right]^n \tag{10}$$

When the diameter of quartz (D_q) is 250 μm, the corresponding zircon grain size (D_z) is 158 μm. In other words, under the same hydrodynamic conditions, when a 250 μm quartz grain initiates settling, a 158 μm zircon grain simultaneously begins to settle.

Therefore, under identical hydrodynamic transport conditions, zircon grains exhibit equivalent behavior to 250 μm quartz grains at two critical size thresholds: 113 μm (initial motion) and 158 μm (hydraulic settling). While these hydrodynamic models assume idealized conditions, they remain consistent with empirical observations (Fig. 6 and Supplementary Fig. S7) that identify 150 μm as the critical threshold distinguishing between sea ice-rafted and iceberg-rafted zircon transport.

S4. Surface microfeature of quartz grains

Sediment transport processes, such as flowing water, wind, and glaciers, as well as different depositional environments, leave mechanical or chemical traces on the surface of quartz grains[33]. Scanning electron microscope (SEM) secondary electron imaging of quartz grain surfaces can help identify these tiny structures and morphologies, referred to as "microfeatures"[33,34]. Previous studies suggest that quartz grains in sediments derived from the Quaternary ice sheets are mostly subangular and exhibit numerous mechanical features, including conchoidal fractures, step-like fractures, subparallel linear fractures, scratches, and breakage blocks/layers, which are primarily formed through the abrasion and crushing action of thick glaciers[35-41]. In contrast, quartz grains in modern sea ice sediments are mostly subrounded and lack mechanical microfeatures[42,43].

To determine whether the sandy components in our samples were derived from sea-ice entrainment or from glacial erosion, we obtained SEM secondary electron images of coarse-grained quartz (see Materials and methods) from one Holocene (sea ice-borne) sample from core LV90-8-1, as well as five samples from sand-rich IRD layers in cores LV90-8-1 and LV90-9-1, including late GP 1, late IGP 2, post GP 2, late GP 4, and late GP 6 (Supplementary Fig. S9). These sand-rich IRD layers correspond to the sampling horizons of detrital U-Pb zircon (Fig. 3). Surface microfeatures were identified using the classification and interpretation model of Immonen (*36*), which was developed based on a synthesis of previous studies. This model, widely applied to Arctic sediments, categorizes microfeatures into four main groups: roundness, relief, mechanical features, and chemical features (Supplementary Fig. S9).

Our results show that in the LV90-8-1 Holocene sample (sea ice-borne), most of the quartz grains are subrounded, with few mechanical features (Supplementary Figs. S8A, S8B and S9). In contrast, the quartz grains in the sand-rich IRD samples from LV90-8-1 and LV90-9-1 are predominantly subangular (Figs. S8C and S8D) and exhibit a range of mechanical features, such as subparallel linear fractures, step-like fractures (Figs. S8C-F), breakage blocks or layers (Figs. S8G and S8H), fractured planes (Figs. S8I and S8J), edge abrasion (Fig. S8K), and crescentic gouges (Fig. S8L). Approximately 78–83% of the quartz grains in the sand-rich IRD samples show one or more mechanical features, compared to only 26% in the Holocene sea ice-borne sample (Fig. S9 and Source data S4).

Statistical analysis of quartz surface microfeatures in modern subglacial till deposits reveals notable consistencies with our sand-rich IRD samples[44]. These glacial signatures predominantly include crushing-induced features (subparallel linear fractures, step-like fractures, conchoidal fractures, fractured planes, troughs, grooves, crescentic gouges) and abrasion features (edge

abrasion)[37]. However, the frequency distributions of these features show regional variations. For example, studies by Mahaney et al. (*45*) indicate that subparallel linear fractures are predominant (>40% occurrence) in Antarctic tills, whereas step-like features are dominant in North American tills[46] and Norwegian tills[47]. Greenland tills are characterized by high frequencies of edge abrasion[48]. This spatial variability may result from either analytical subjectivity or distinct subglacial conditions across different regions[45]. Notably, all diagnostic glacial microfeatures identified in tills are also present in our sand-rich IRD samples (Supplementary Fig. S9). Critical indicators, including subparallel linear fractures, step-like fractures, and fractured planes, account for over 30% of occurrences in the IRD samples, providing robust microtextural evidence for glacial influences on these deposits (Supplementary Fig. S9).

Although angular shapes are important indicators for identifying subglacial environments[37], quartz grains from subglacial settings can also exhibit subrounded forms. For example, only 30–60% of quartz grains from Antarctic tills possess sharp edges[44], and around 20% of quartz grains in Norwegian tills are subrounded[47]. The formation of subrounded quartz in subglacial environments may result from edge abrasion[49]. Additionally, many quartz grains in tills undergo weathering, which is demonstrated by a higher proportion of silicate precipitation (~20-40% in Antarctic tills, ~50% in North America, and ~50-80% in Greenland tills)[45-47]. Therefore, the presence of subrounded quartz grains (15–30%) and silicate precipitation (over 40%) in sand-rich IRD samples does not exclude the possibility that these grains are of glacial origin (Figs. S8M-Q and S9).

However, mechanical features in IRD are not exclusive to subglacial environments. Woronko et al. (*50*) suggested that features such as conchoidal fractures, breakage blocks, fracture planes, and crescentic gouges can also form in periglacial environments, as a result of freeze-thaw

processes involving ice. Nevertheless, they noted that quartz grains from periglacial environments rarely show step-like fractures or subparallel linear fractures, indicating that these features are characteristic of subglacial conditions. Our analysis of the sand-rich layers reveals that over 60% of the quartz grains exhibit step-like fractures and subparallel linear fractures ("Subglacial features" in Supplementary Fig. S9), suggesting that at least 60% of the quartz grains originate from subglacial environments. However, the absence of these subglacial features in other quartz grains does not rule out a glacial origin, as it is difficult to distinguish these grains from those originating in periglacial environments. Moreover, quartz grains from subglacial environments are often significantly modified by subsequent transport processes. For example, quartz grains transported by glacial meltwater after glacial erosion show a gradual reduction in mechanical features with increasing transport distance[51]. Therefore, the mechanical features of quartz grains in sand-rich IRD samples likely have not undergone extensive modification during long-distance fluvial transport.

S5. MATLAB code for kernel density estimate (KDE) estimation.

```
function [density] = calculateKDEwithBw(data, x_min, x_max, num_points, bandwidth)
% This function calculates a kernel density estimate (KDE) with an adjustable bandwidth for a
given dataset. It evaluates the density over a specified range of x-values and includes steps for
adjusting the bandwidth based on the pilot density estimate. The final output includes the KDE
values and visualizes the results, including marking any local peaks.
% Inputs:
    %   data - Dataset (vector of values)
```

```
    %   x_min - Minimum value on the x-axis
    %   x_max - Maximum value on the x-axis
    %   num_points - Number of evaluation points on the x-axis
    %   bandwidth - Bandwidth parameter for KDE
% Output:
    %   density - Kernel density estimate values corresponding to the x-axis values

    % Generate x-axis values over the specified range
    x_vals = linspace(x_min, x_max, num_points);

    % Number of data points
    n = length(data);

    % Initialize the density array
    density = zeros(size(x_vals));

    % Compute pilot density (pdens)
    pdens = zeros(size(data));
    for i = 1:n
        % Compute the kernel density around each data point using a very small range
        pdens(i) = mean(calculateKDE(data, data(i)-1e-10, data(i)+1e-10, 2, 15));
    end

```

```
% Compute G value: Extract positive values from pdens and compute the geometric mean
fpos = pdens(pdens > 0);  % Extract positive values
N = length(fpos);  % Number of positive values
G = exp(sum(log(fpos)) / N);  % Compute the geometric mean

% Compute the kernel density estimate
for i = 1:n
    % Compute the adjusted bandwidth
    lambda = sqrt(G / pdens(i));
    % Use the adjusted bandwidth to compute the kernel density
    density = density + exp(-0.5 * ((x_vals - data(i)) / (bandwidth * lambda)).^2) / (sqrt(2 * pi) * (bandwidth * lambda));
end

% Normalize the density values
density = density / n;

% Filter the x-values and density values within the integration range (75 to 125)
mask = (x_vals >= 75) & (x_vals <= 125);  % Select indices that fall within the range
x_selected = x_vals(mask);  % Extract x-values within the range
density_selected = density(mask);  % Extract density values within the range

% Perform numerical integration using the trapezoidal rule
```

```
    integral_result = trapz(x_selected, density_selected);

    % Combine x-values and density values into a n x 3 matrix
    density = [x_vals(:), density(:)];

    % Display the number of data points (e.g., zircon grains)
    fprintf('Number of zircon grains: %d\n', n);

    % Display the integration result over the selected range
    fprintf('Density integration result from %.2f to %.2f: %.4f\n', 75, 125, integral_result);

    % Find local peaks (local maxima)
    [pks, locs] = findpeaks(density(:, 2)); % Peaks can be adjusted by the 'MinPeakProminence' parameter
    peak_x = density(locs, 1);  % x-coordinates of the peaks

    % Display peak information
    for i = 1:length(pks)
        fprintf('Peak %d: x = %.2f, y = %.4f\n', i, peak_x(i), pks(i));
    end

    % Plot the results
    figure;
```

```
    plot(density(:, 1), density(:, 2), 'LineWidth', 2);
    hold on;
    area(x_selected, density_selected, 'FaceColor', 'r', 'FaceAlpha', 0.5);  % Highlight the integration area
    scatter(peak_x, pks, 100, 'b', 'filled');  % Mark the peaks
    xlabel('x');
    ylabel('Density');
    title('Kernel Density Estimation with Bandwidth Adjustment');
    hold off;
end
```

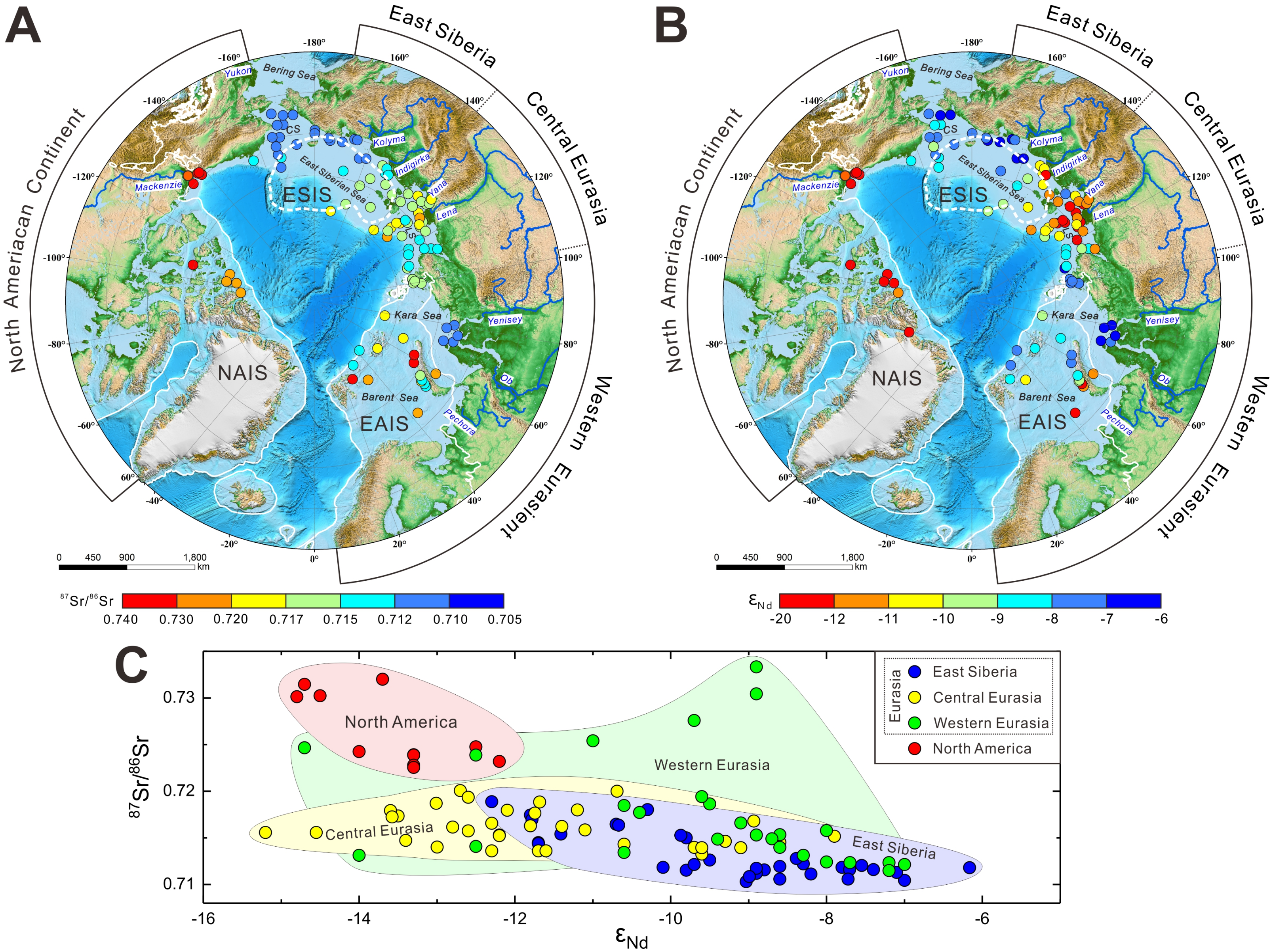

A
B
C
East Siberia
Central Eurasia
Western Eurasient
North Ameriacan Continent
Bering Sea
Yukon
Mackenzie
Kolyma
Indigirka
Yana
Lena
Yenisey
Ob
Pechora
East Siberian Sea
Kara Sea
Barent Sea
ESIS
NAIS
EAIS
CS
-180°
-160°
-140°
-120°
-100°
-80°
-60°
-40°
-20°
0°
20°
40°
60°
80°
100°
120°
140°
160°
0 450 900 1,800 km
$^{87}Sr/^{86}Sr$
0.740 0.730 0.720 0.717 0.715 0.712 0.710 0.705
ε_{Nd}
-20 -12 -11 -10 -9 -8 -7 -6
North America
Western Eurasia
Central Eurasia
East Siberia
Eurasia
East Siberia
Central Eurasia
Western Eurasia
North America
$^{87}Sr/^{86}Sr$
0.73
0.72
0.71
ε_{Nd}
-16 -14 -12 -10 -8 -6

Fig. S1. Sr-Nd isotopic values of surface sediments on the circum-Arctic continental shelves. The $^{87}Sr/^{86}Sr$ (**A**) and ε_{Nd} (**B**) are effective provenance tools in distinguishing sources between North America and the entire Eurasia due to their distinctive values (**C**). The entire Eurasia (including East Siberia, central Eurasia, and western Eurasia) exhibits relatively higher ε_{Nd} (mostly -6 to -14) and lower $^{87}Sr/^{86}Sr$ (mostly 0.71 to 0.72), whereas the North America show lower ε_{Nd} values (mostly < -12) and higher $^{87}Sr/^{86}Sr$ (mostly > 0.72). However, the ranges of Sr-Nd isotopic values of different shelves within the Eurasian shelf were overlapped **(C)**. For example, both the East Siberian area (including the Chukchi Sea and the East Siberian Sea, which covered by proposed ESIS) and the Kara Sea (covered by EAIS) show high ε_{Nd} (mostly -6 to -9) and low $^{87}Sr/^{86}Sr$ (mostly < 0.712), thus Sr-Nd isotopic values cannot be regarded as an exclusive indicator of East Siberian provenance. Epsilon Nd values (ε_{Nd}) were calculated using chondritic values of $^{143}Nd/^{144}Nd = 0.512638$[52]. Data are from Ref. 1, 2 and references therein. Basemap is from the ETOPO Global Relief Model (https://www.ncei.noaa.gov/products/etopo-global-relief-model). ESIS: East Siberian Ice Sheet[53] (enclosed by the white dashed line); NAIS: North American Ice sheet[54]; EAIS: Eurasian Ice Sheet[54]; CS = Chukchi Sea; LS = Laptev Sea.

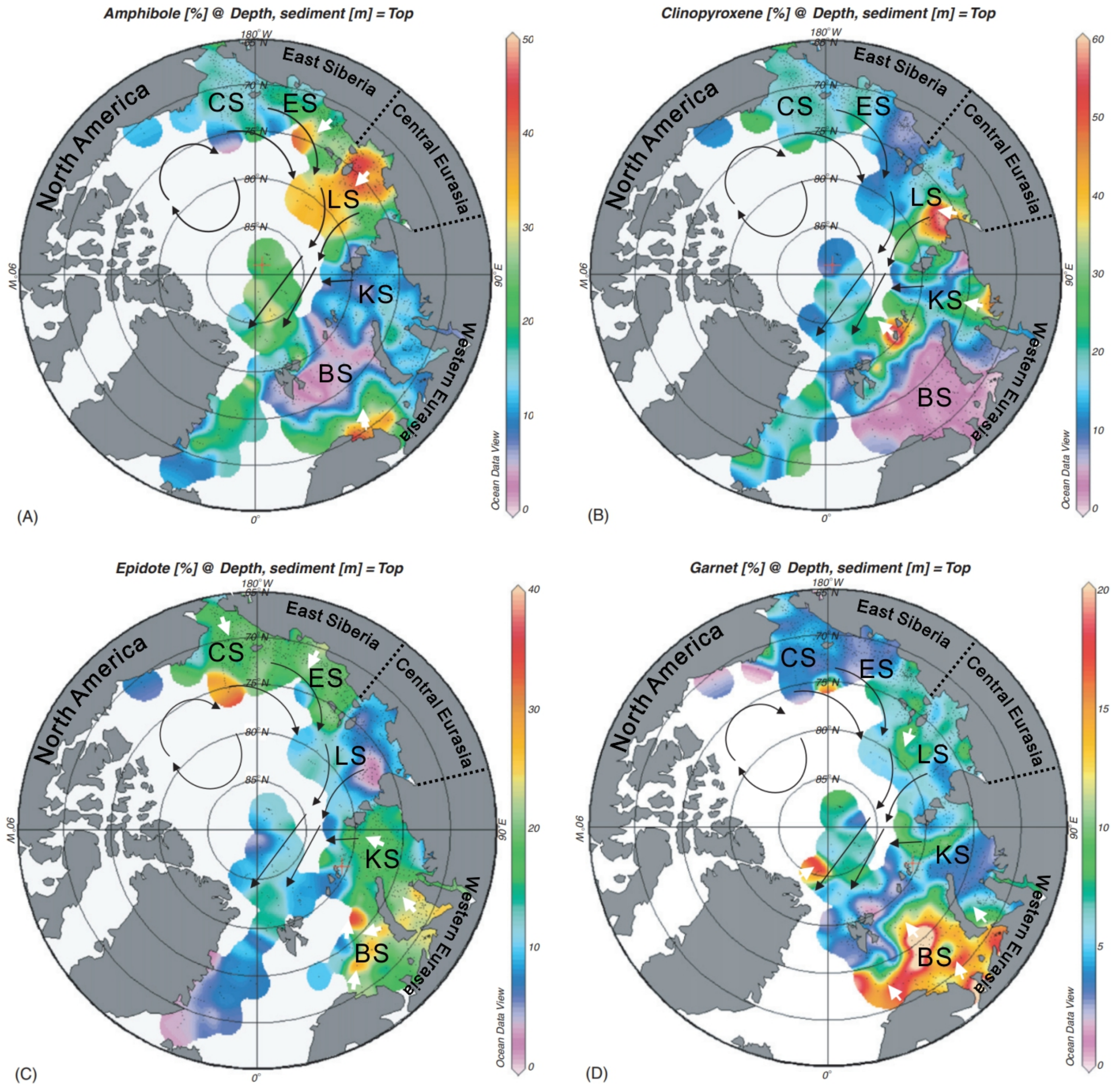

Fig. S2. Heavy-mineral distribution in Arctic Ocean surface sediments. Redrawn from Ref. 3. Note that none of the heavy mineral types in the sediment of East Siberian area exceed 30%, thus no heavy mineral can be used to represent its source. CS = Chukchi Sea; ES = East Siberian Sea; LS = Laptev Sea; KS = Kara Sea; BS = Barents Sea.

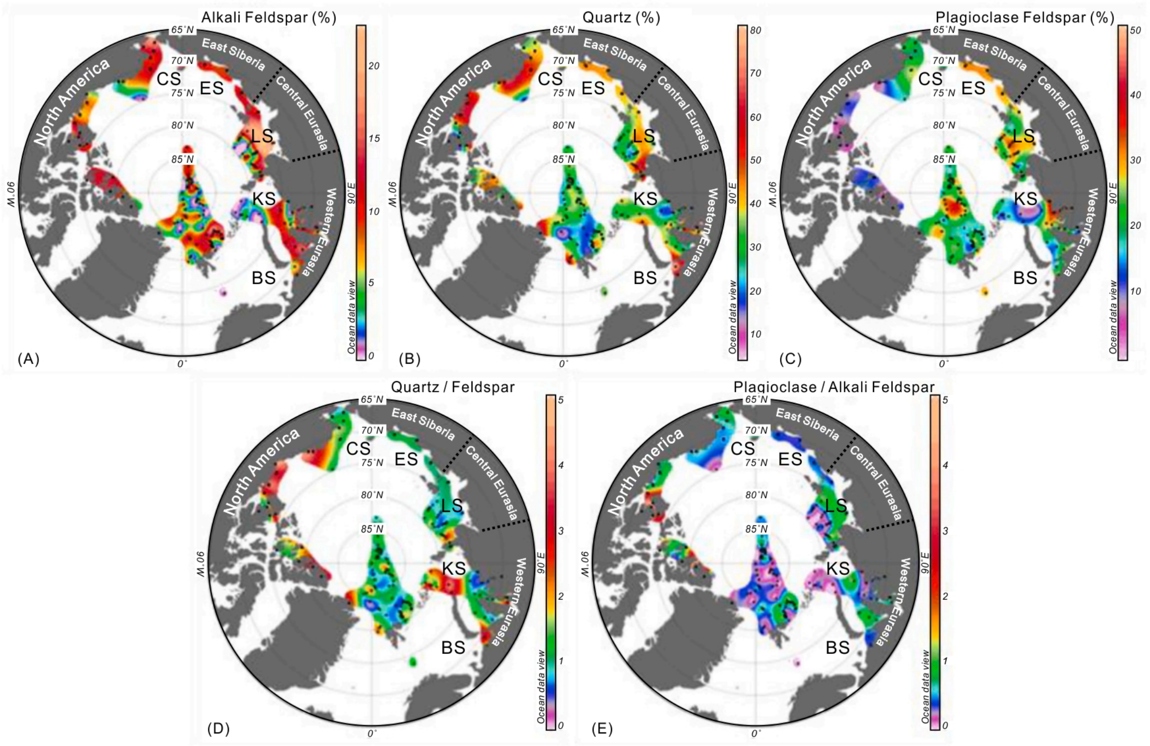

Fig. S3. Quartz and feldspar distribution in Arctic Ocean surface sediments. Redrawn from Ref. 4. Note that the quartz and feldspar contents in East Siberia are similar to those in central Eurasia (Laptev Sea) and western Eurasia (Kara Sea). CS = Chukchi Sea; ES = East Siberian Sea; LS = Laptev Sea; KS = Kara Sea; BS = Barents Sea.

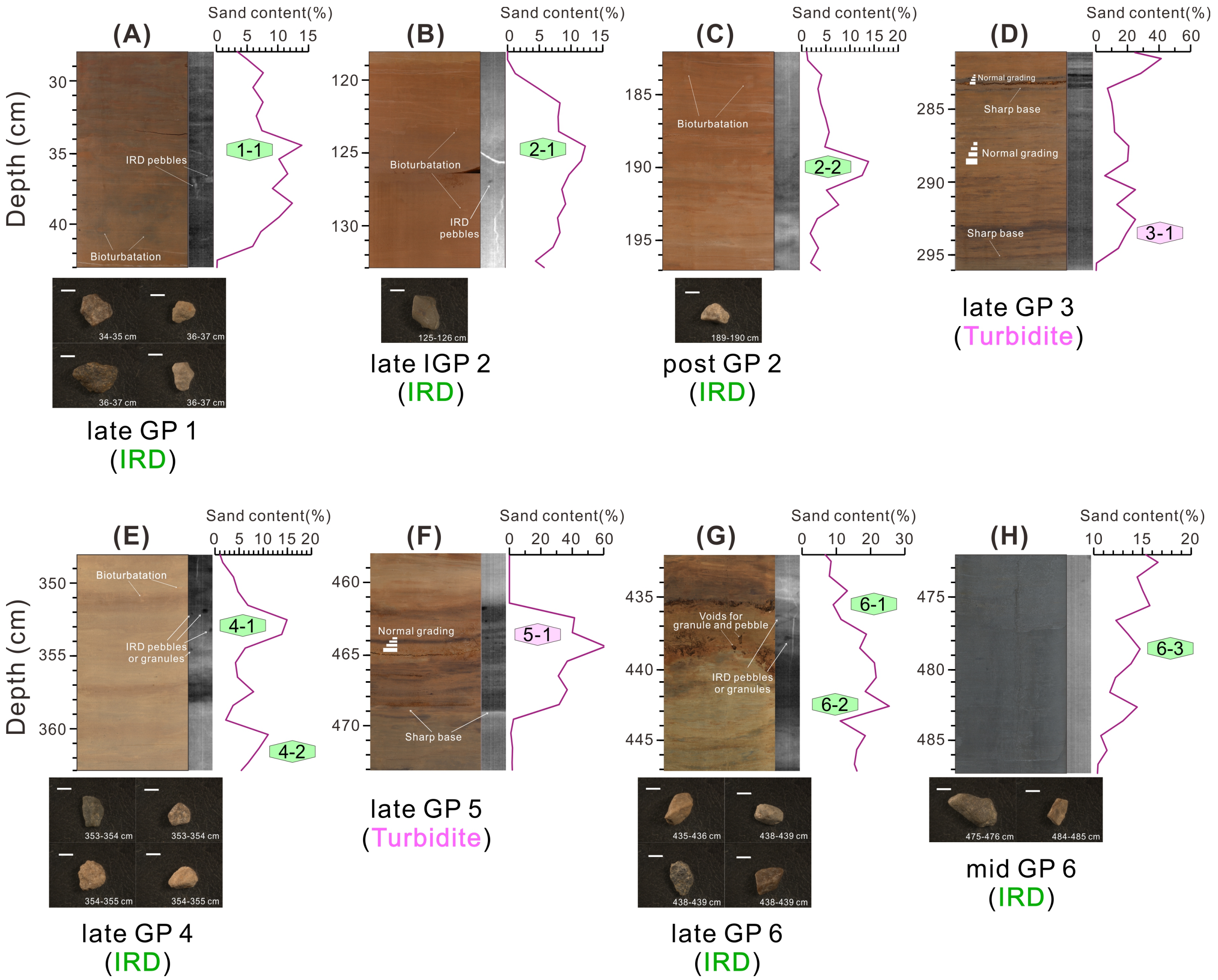

(A)
(B)
(C)
(D)
(E)
(F)
(G)
(H)
Sand content(%)
Depth (cm)
IRD pebbles
Bioturbatation
1-1
IRD pebbles
2-1
Bioturbatation
2-2
Normal grading
Sharp base
3-1
4-1
4-2
IRD pebbles or granules
5-1
6-1
6-2
6-3
Voids for granule and pebble
IRD pebbles or granules
late GP 1 (IRD)
late IGP 2 (IRD)
post GP 2 (IRD)
late GP 3 (Turbidite)
late GP 4 (IRD)
late GP 5 (Turbidite)
late GP 6 (IRD)
mid GP 6 (IRD)

Fig. S4. Core photo, X-ray radiograph and sand content of typical sand-rich layers in core LV90-8-1 (A-F) and LV90-9-1 (G, H). Sample positions of detrital zircon U-Pb dating are marked by hexagon with sample name below. Images of granules and pebbles found in the samples with positions are shown below. The white error bar represents 2 mm. (**A, B, C, E**) Homogeneous composition with bioturbation (mottling); The gradational contacts between adjacent beds indicate continuous deposition of sediments, in which sands are typical ice rafted debris (IRD); (**D, F**) Thin lamination with normal-graded sequence and sharp base indicating turbidite deposition. (**G**) Voids left by fallen granules and pebbles indicate IRD deposition. (**H**) Homogeneous composition with no bioturbation or beddings. GP = Glacial period. IGP = Interglacial period.

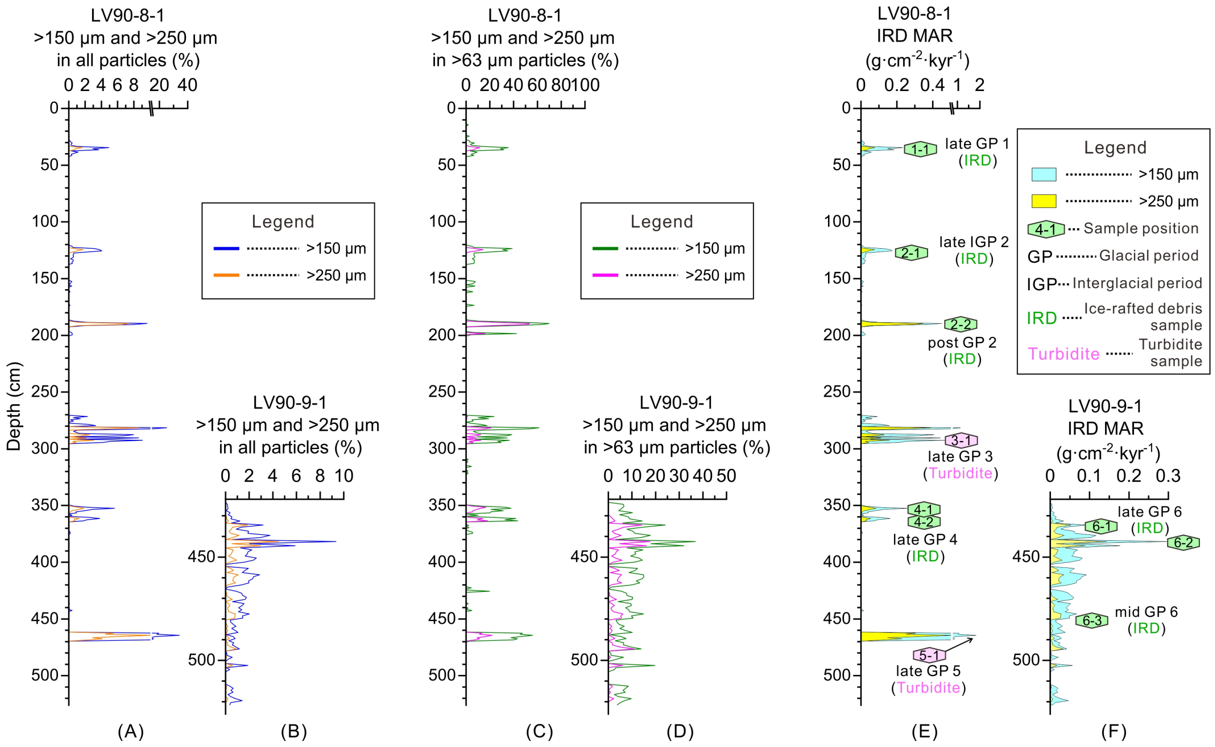

Fig. S5. Content and mass accumulation rate of coarse sand grains in core LV90-8-1 and LV90-9-1. (**A, B**) Proportion of >150 μm and >250 μm grains in all particles. (**C, D**) Proportion of >150 μm and >250 μm grains in all sand particles (>63 μm). (**E, F**) Mass accumulation rate (MARs) of >150 μm and >250 μm grains. The calculation process of MARs is detailed in the Methods. Detrital zircon dating samples are marked in E and F, same as Fig. 3.

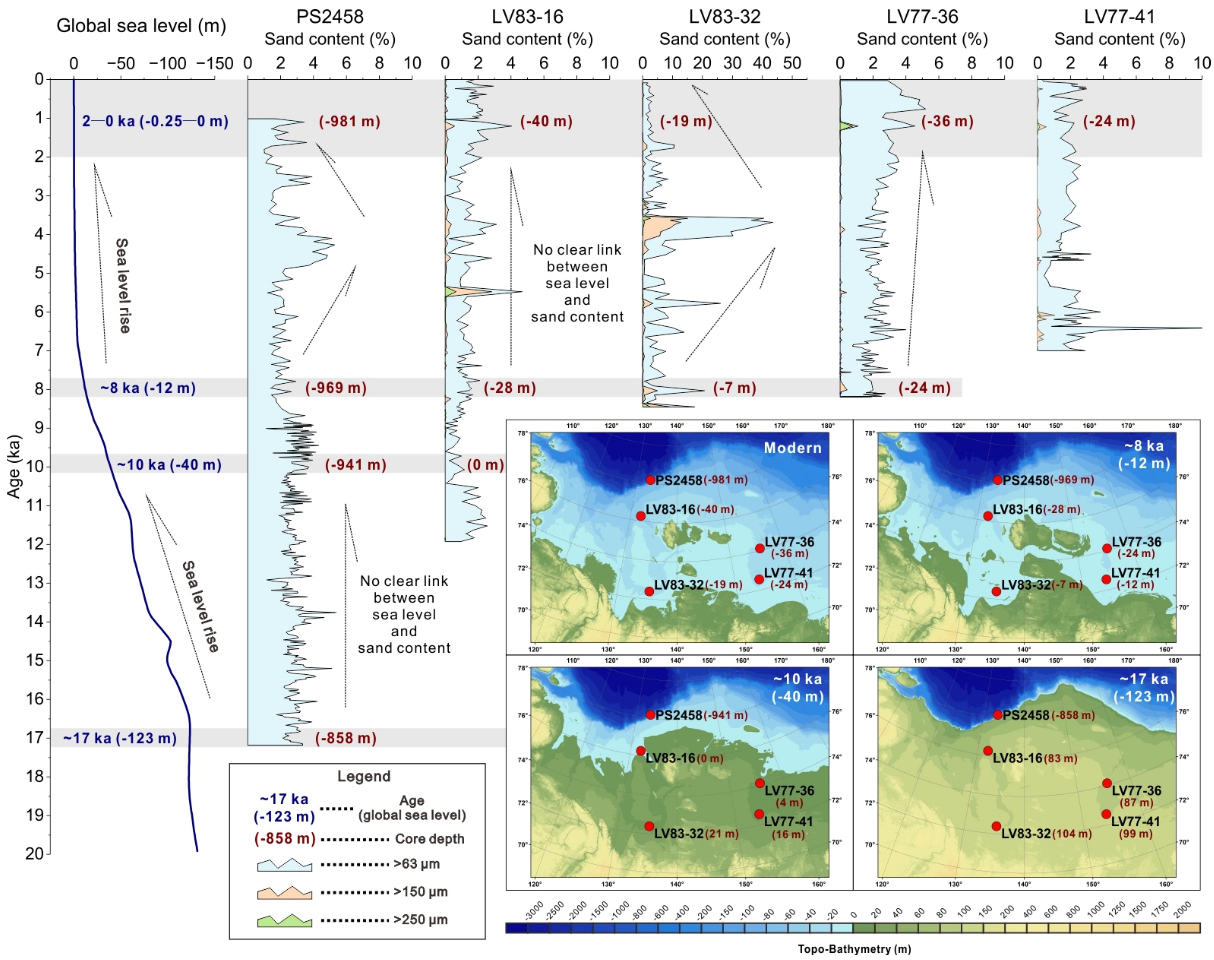

Fig. S6. Sand content (>63 μm, >150 μm and >250 μm) in cores from the continental shelves of the East Siberian Sea and Laptev Sea, showing no clear relationship with sea level rise since ~17 ka. Four time slices (~17 ka, 10 ka, 8 ka, and 2–0 ka) are shaded, with corresponding sea levels and core depths indicated in parentheses. The inset shows the core locations and topo-bathymetric maps at each time slice. The sand content of core PS2458[55] remained stable during rapid sea-level rise (~17–9 ka) with no significant decrease during the highstand period (~6–0 ka). The sand content of core LV83-16[56] stayed consistently low since 12 ka, except for a >150 μm peak at ~5.5 ka. The core LV83-32[56] exhibited a distinct sand content peak around 4 ka. The sand content of core LV77-36[57] showed a gradual increase since ~8 ka. The core LV77-41[57] maintained low sand content levels since ~7 ka. Topographic and bathymetric data are from the GEBCO Grid (https://www.gebco.net/data-products/gridded-bathymetry-data).

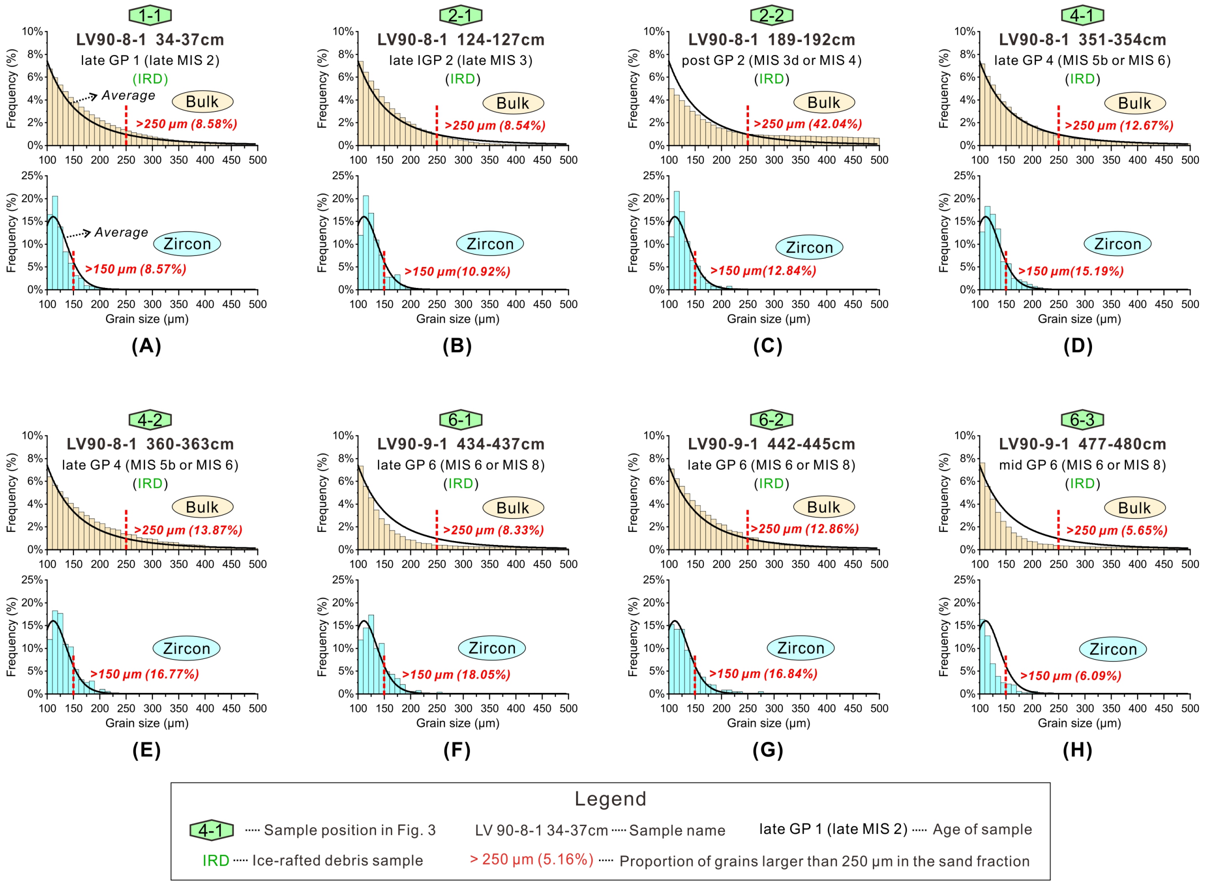

Fig. S7. Grain size distribution of bulk and zircon grains from the sand-rich IRD samples in cores LV90-8-1 and LV90-9-1. The vertical scale shows frequency per 10 μm bin normalized to total sand fraction (>63 μm). The black curve represents the fitted average grain size distribution for all sand-rich IRD samples shown in Fig. 5 and Fig. 6. See raw data in Supplementary Data S2 and S3.

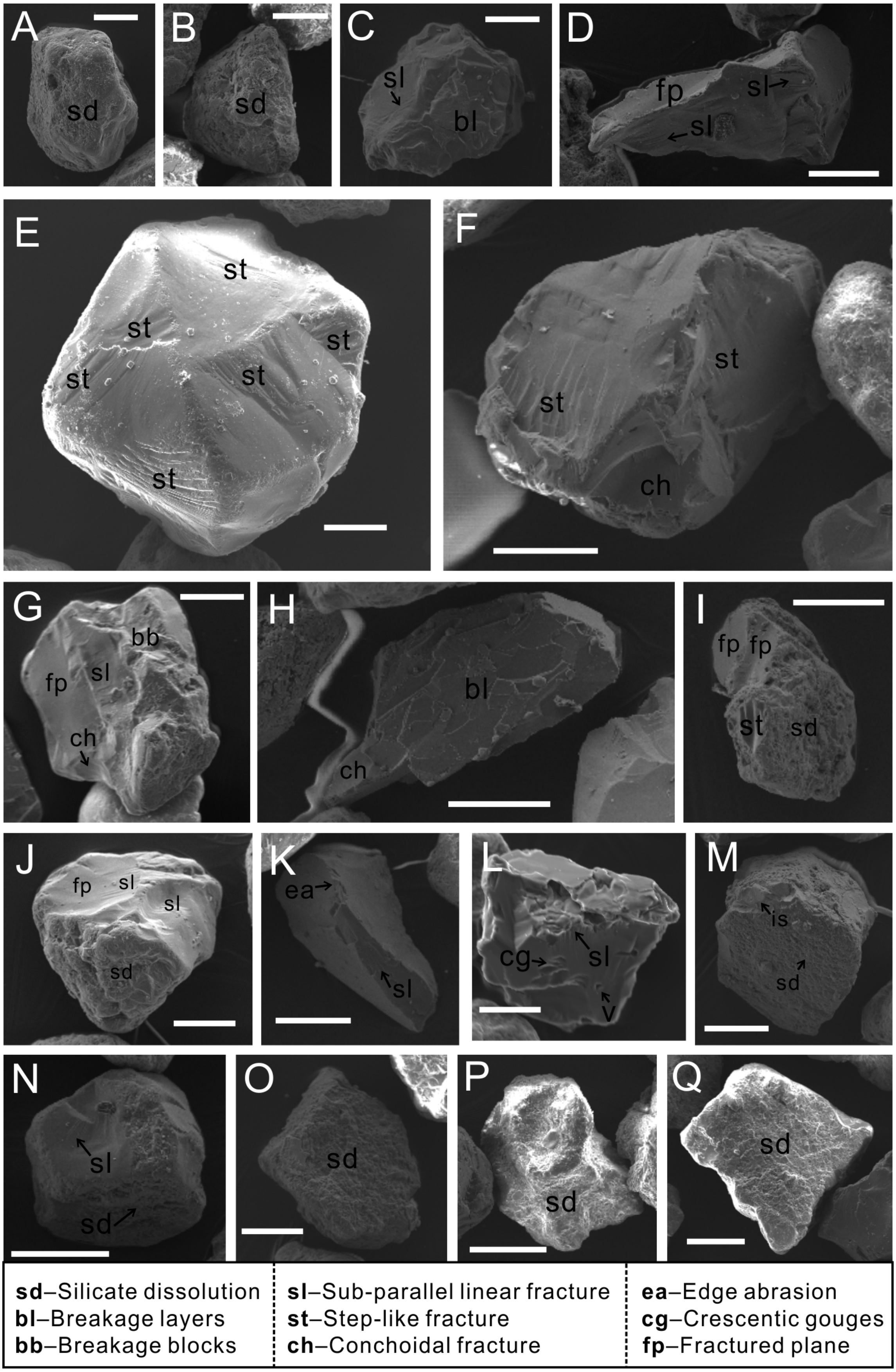
A
sd
B
sd
C
sl
bl
D
fp
sl
sl
E
st
st
st
st
st
st
F
st
st
ch
G
bb
fp
sl
ch
H
bl
ch
I
fp fp
st
sd
J
fp
sl
sl
sd
K
ea
sl
L
cg
sl
v
M
is
sd
N
sl
sd
O
sd
P
sd
Q
sd
sd–Silicate dissolution
bl–Breakage layers
bb–Breakage blocks
sl–Sub-parallel linear fracture
st–Step-like fracture
ch–Conchoidal fracture
ea–Edge abrasion
cg–Crescentic gouges
fp–Fractured plane

Fig. S8. Example SEM secondary electron photos of quartz grain surface features for Holocene sea ice-borne sample (A, B) and sand-rich IRD samples (C-Q) of core LV90-8-1. (**A, B)** Subrounded grain with heavy silicate dissolution; **(C)** Subangular grain with breakage layers and sub-parallel linear fractures; (**D)** Subangular grain with sub-parallel linear fractures and fractured plane; (**E, F)** step-like fracture and conchoidal fracture; **(G)** Breakage blocks, sub-parallel linear fractures, fractured plane and conchoidal fracture; **(H)** Breakage layers and conchoidal fracture; **(I)** Fractured plane, step-like fracture and silicate dissolution; **(J)** Fractured plane, sub-parallel linear fractures and silicate dissolution; **(K)** Edge abrasion and sub-parallel linear fractures; **(L)** Impacted v-point, crescentic gouges and sub-parallel linear fractures; **(M, N)** Fractures after silicate dissolution; **(O-Q)** Subangular grain with heavy silicate dissolution. Scale bar = 50 μm.

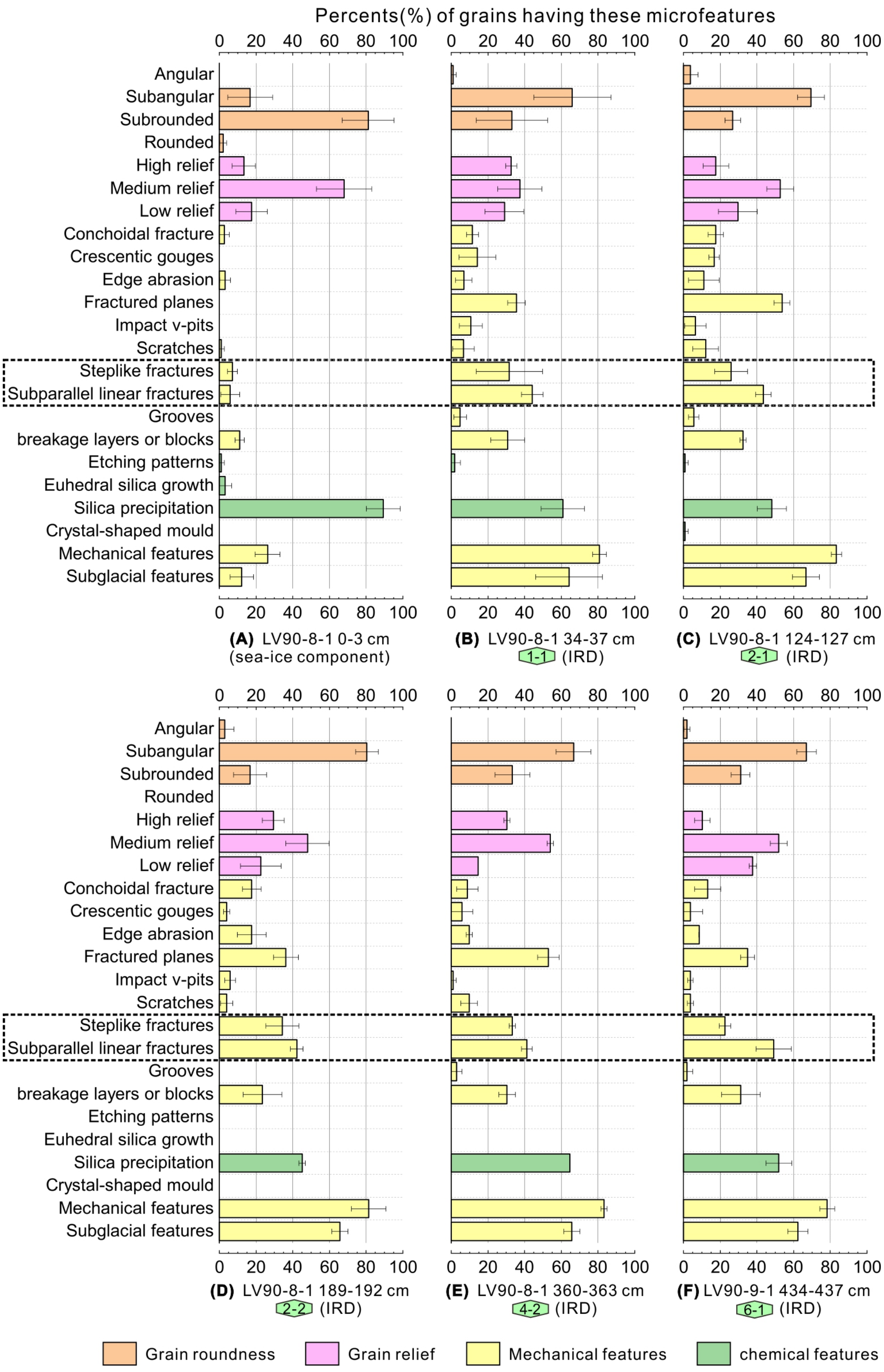

Fig. S9. Microfeature percentage of quartz grains in sandy samples from Holocene sediments (A) as well as sand-rich IRD layers (B-F) of core LV90-8-1 and LV90-9-1. Error bars represent 1 SD (Standard Deviation), calculated from the analysis of three subsamples. Features affected solely by glaciation (subglacial features) are framed with dotted lines. Note that quartzs in Holocene samples have fewer mechanical features and subglacial features than those in sand-rich IRD samples. See raw counting data in Supplementary data S4.

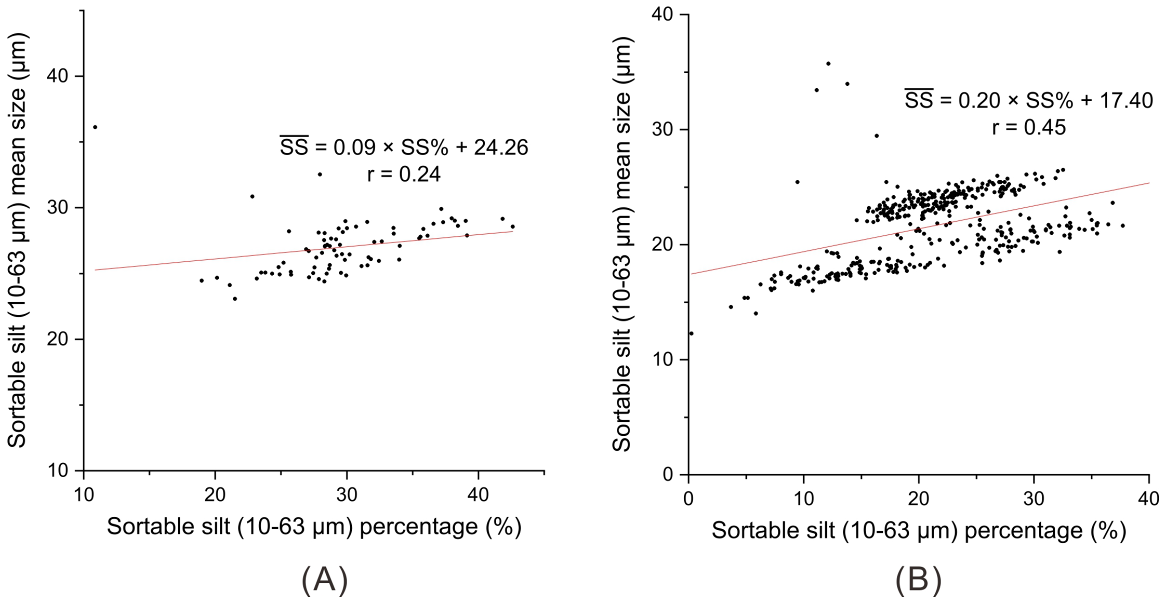

Fig. S10. Correlation between the mean grain size of sortable silt ($\overline{SS}$, 10–63 μm) and its proportion within the <63 μm fraction (SS%) in the sediments from LV90-8-1. (A) Sediments from sand-rich IRD layers; (B)Sediments excluding the sand-rich IRD layers. Original data can be found in Supplementary data S3.

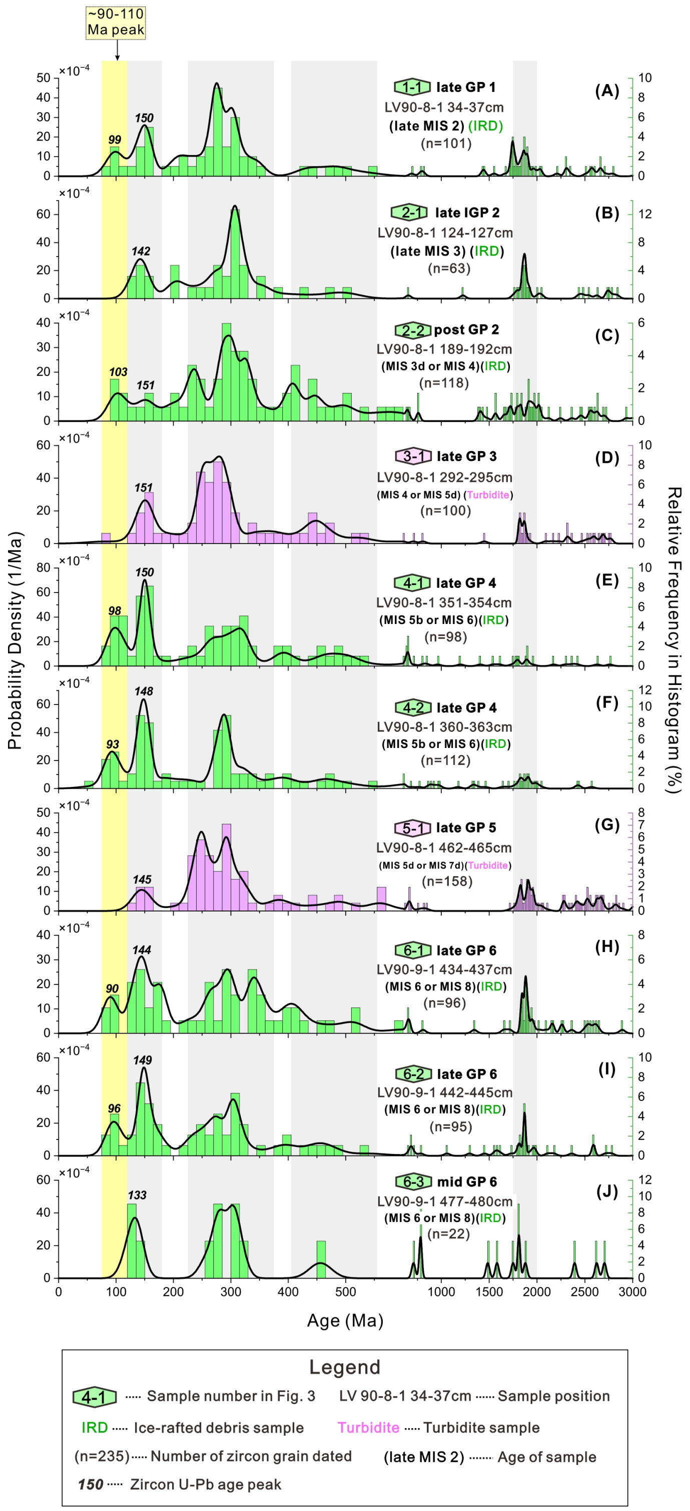

Fig. S11. Detrital zircon U–Pb age distributions of >150 µm particles in sand-rich layers from cores LV90-8-1 and LV90-9-1. Sample positions are marked in Fig. 3. Zircon U-Pb age peaks of ~90-110 Ma, ~140-160 Ma, ~220–360 Ma, 420–560 Ma and ~1750–2000 Ma are shaded in the zircon age distribution. The age peak of ~90-110 Ma occurs in the IRD samples of late GP 1 (**A**), post GP 2 (**C**), late GP 4 (**E, F**) and late GP 6 (**H, I**), consistent with the pattern observed in the >63 µm zircon fraction (Fig. 4).

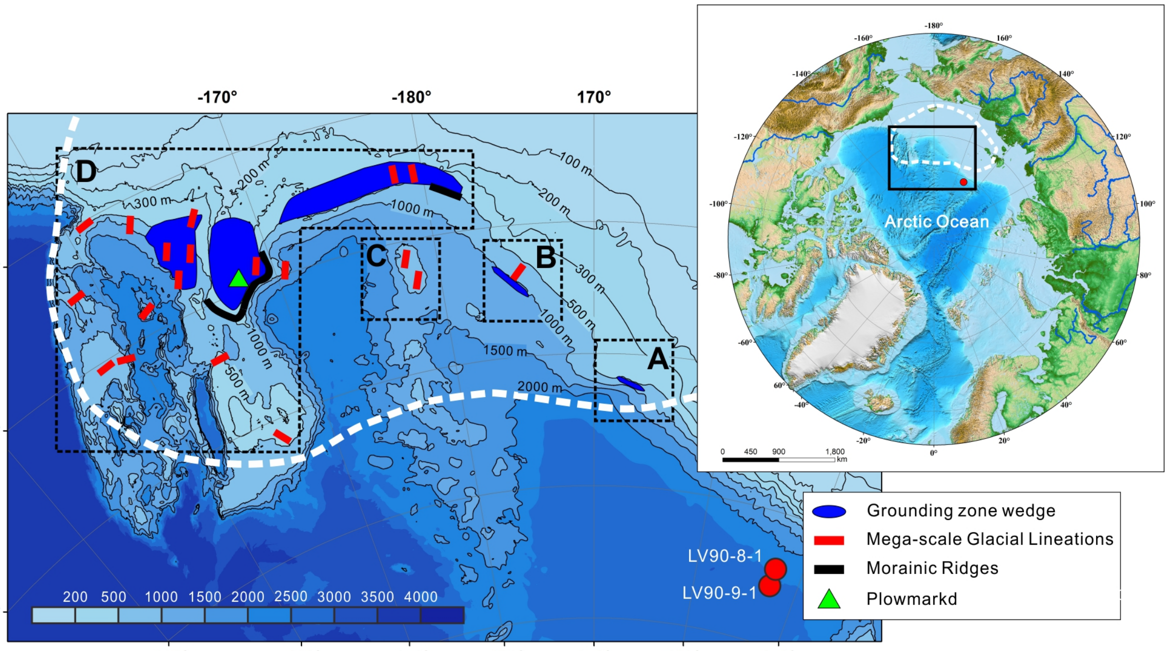

Fig. S12. Glacial landforms identified on the East Siberian continental margin. (A) Grounding zone wedges (GZWs) within the De Long glacial trough[58]; (B) GZWs and Mega-scale Glacial Lineations (MSGLs) on the East Siberian Sea continental margin[53]; (C) MSGLs on the Arliss Plateau[53]; (D) GZWs, MSGLs, Morainic Ridges and ice-originated plowmarks on the Chukchi Sea continental margin and Chukchi Borderland[53,58-64]. White dashed line shows the proposed extent of the East Siberian Ice Sheet[53]. Bathymetric data are from the GEBCO Grid (https://www.gebco.net/data-products/gridded-bathymetry-data) and basemap of the thumbnail is from the ETOPO Global Relief Model (https://www.ncei.noaa.gov/products/etopo-global-relief-model).

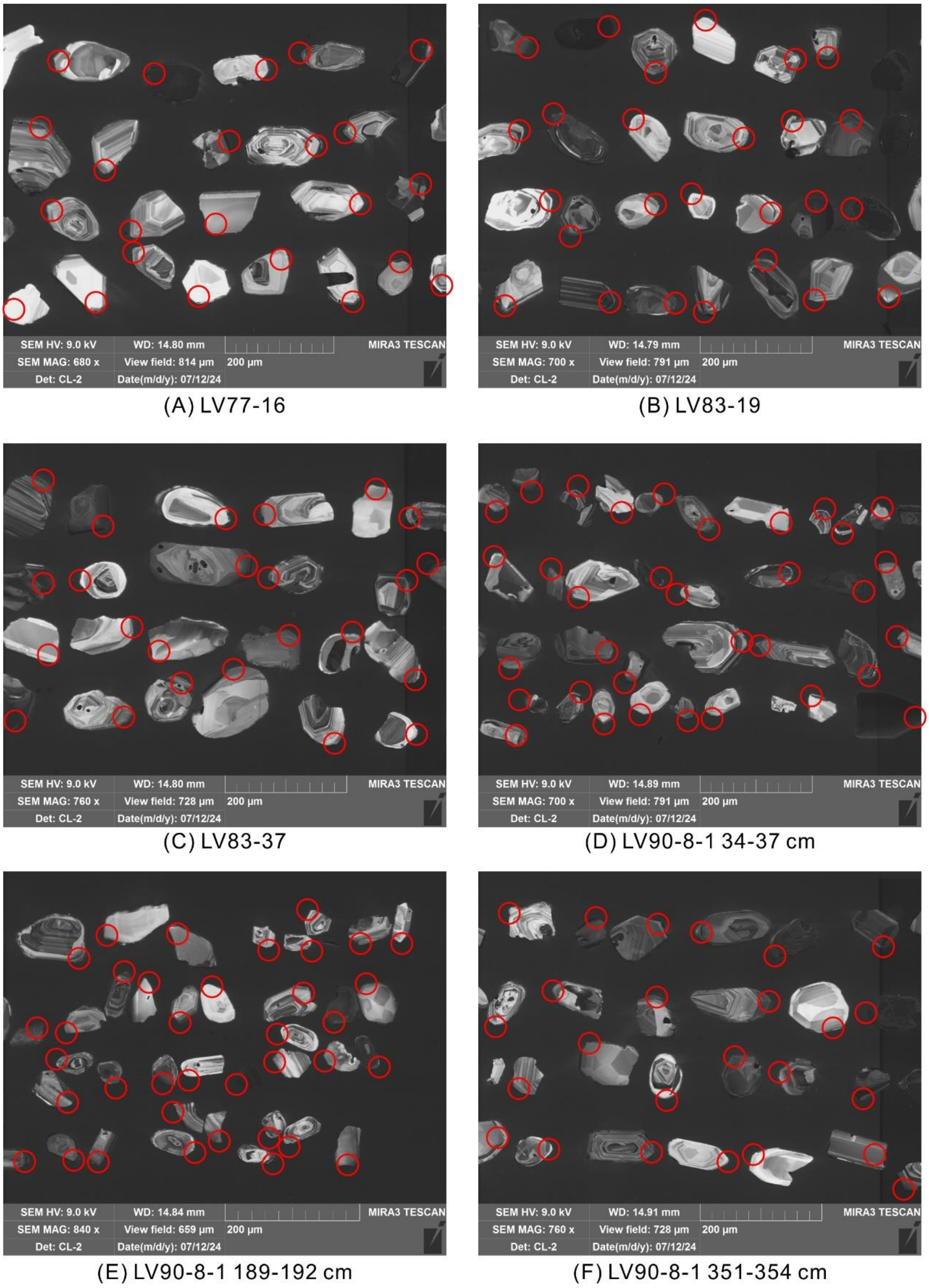

Fig. S13. Example of cathodoluminescence (CL) images of zircon grains from samples from surface (A-C) and core LV90-8-1 (D-F). Red circles with a diameter of 37 µm represent the laser ablation points, which are chosen to avoid the xenocrystic core, voids and inclusions based on the CL images.

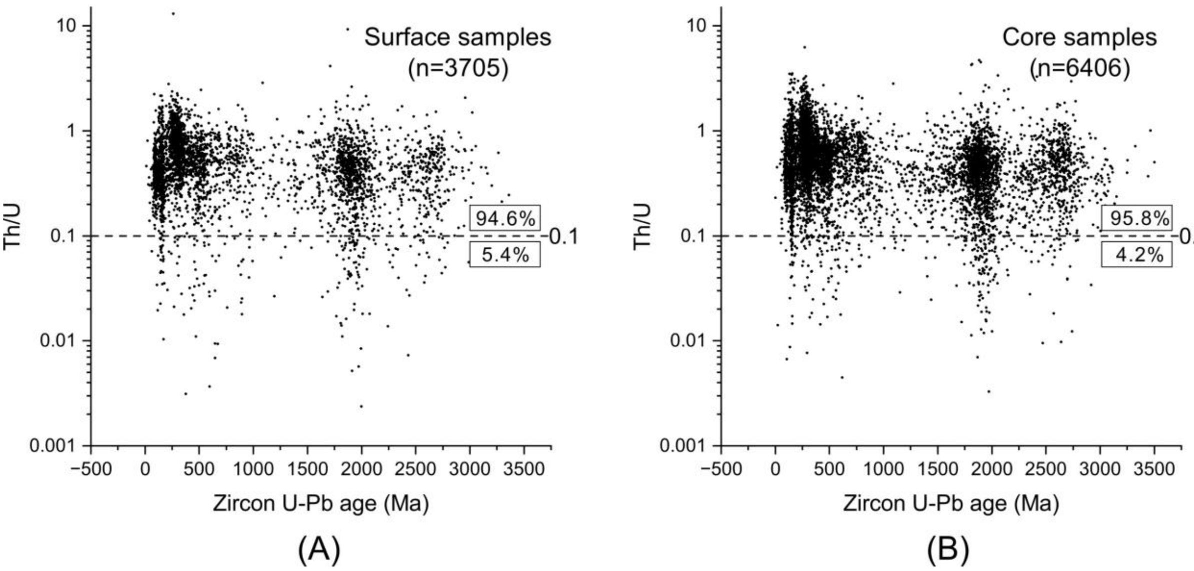

Fig. S14. Th/U ratio of zircon grains in our surface (A) and core (B) samples showing most of the zircon grains (~95 %) are of magmatic origin (Th/U > 0.1)[12].

Table S1. Sampling information of surface samples on Eurasian continental shelves and cores LV90-8-1 and LV90-9-1.

Sample	Longitude (°E)	Latitude (°N)	Water depth (m)	Cruise	Platform	Time
**Surface sample**						
LV77-07	-173.5	71.18	43	LV77	R/V "Academician M.A. Lavrentiev"	2016
LV77-12	174.36	70.73	37			
LV77-16	166.04	70.09	15			
LV77-19	162.55	72.14	22			
LV77-21	167.49	74.12	43			
LV77-31	161.31	77.24	94			
LV77-33	159.26	75.86	46			
LV77-34	158.5	75.25	36			
LV83-10	142.32	78.66	81	LV83	R/V "Academician M.A. Lavrentiev"	2018
LV83-17	132.39	76.42	48			
LV83-19	124.85	74.59	38			
LV83-26	130.85	72.36	11			
LV83-32	135.33	72.39	19			
LV83-37	151.72	72.99	11			
AMK78-18	104.23	77.95	250	AMK78	R/V "Akademik Mstislav Keldysh"	2019
AMK78-20	73.34	73.58	26			
ARC9-C24	-161.53	71.71	38	ARC9	R/V "Xuelong"	2018
**Gravity core**						
LV90-8-1	152.50	80.72	2300	LV90	R/V "Academician M.A. Lavrentiev"	2020
LV90-9-1	152.46	80.98	2546			

Table S2. Proportion of ~90-110 Ma peak in the detrital zircon age distribution from samples from surface of Eurasian continental shelves and cores LV90-8-1 and LV90-9-1.

Sample name	Total zircon numbers	Proportion of ~90-110 Ma age peak[*]
**Surface sample**		
Pechora River Mouth[65]	185	/[†]
Ob River Mouth[66]	401	/
Ob River Mouth[65]	160	/
Yenisey River Mouth[65,66]	220	/
AMK78-20	207	/
AMK78-18	275	/
LV83-19	235	/
LV83-17	205	/
LV83-26	205	/
LV83-32	201	/
LV83-10	200	/
LV83-37	228	/
LV77-34	205	3.26%
LV77-33	207	11.08%
LV77-31	200	4.64%
LV77-21	233	8.27%
LV77-19	236	10.70%
LV77-16	234	29.93%
LV77-12	236	34.84%
LV77-07	204	11.67%
ARC9-C24	194	9.04%
Yukon River Mouth[67]	200	30.48%
**Core sample**		

LV90-8-1 34-37 cm—late GP 1 (IRD)	1181	2.22%
LV90-8-1 124-127 cm—late IGP 2 (IRD)	577	/
LV90-8-1 189-192 cm—post GP 2 (IRD)	981	2.27%
LV90-8-1 292-295 cm—late GP 3 (Turbidite)	412	
LV90-8-1 351-354 cm—late GP 4 (IRD)	645	7.83%
LV90-8-1 360-363 cm—late GP 4 (IRD)	668	7.98%
LV90-8-1 462-465 cm—late GP 5 (Turbidite)	485	/
LV90-9-1 434-437 cm—late GP 6 (IRD)	532	4.67%
LV90-9-1 442-445 cm—late GP 6 (IRD)	564	4.01%
LV90-9-1 477-480 cm—mid GP 6 (IRD)	361	/

[*] The proportion of the ~90-110 Ma age peak is calculated by integrating the area within the 90-110 Ma range on the KDE curve.

[†]Age distributions without 90-110 Ma age peak (Fig. 2).

REFERENCES

1. Maccali, J., Hillaire-Marcel, C. & Not, C. Radiogenic isotope (Nd, Pb, Sr) signatures of surface and sea ice-transported sediments from the Arctic Ocean under the present interglacial conditions. *Polar Res.* **37**, 1442982 (2018).

2. Li, Q. et al. Sr, Nd, and Pb isotope provenance of surface sediments on the East Siberian Arctic Shelf and implications for transport pathways. *Chem. Geol.* **618**, 121277 (2023).

3. Stein, R. Chapter Five Modern Environment and Its Record in Surface Sediments. In: Stein, R., editor. *Developments in Marine Geology*: Elsevier; 2008. pp. 247-286.

4. Myers, W. B. & Darby, D. A. A compilation of the silt and clay mineralogy from coastal and shelf regions of the Arctic Ocean. *Mar. Geol.* **454**, 106948 (2022).

5. Bischof, J. F. & Darby, D. A. Mid- to Late Pleistocene ice drift in the western Arctic Ocean: evidence for a different circulation in the past. *Science*. **277**, 74-78 (1997).

6. Darby, D. A., Myers, W., Herman, S. & Nicholson, B. Chemical Fingerprinting, A precise and efficient method to determine sediment sources. *J. Sediment. Res.* **85**, 247-253 (2015).

7. Darby, D. & Bischof, J. F. A Statistical Approach to Source Determination of Lithic and Fe Oxide Grains: An Example from the Alpha Ridge, Arctic Ocean. *J. Sediment. Res.* **66**, 599-607 (1996).

8. Darby, D. A., Ortiz, J. D., Grosch, C. E. & Lund, S. P. 1,500-year cycle in the Arctic Oscillation identified in Holocene Arctic sea-ice drift. *Nat. Geosci.* **5**, 897-900 (2012).

9. Tripati, A. & Darby, D. Evidence for ephemeral middle Eocene to early Oligocene Greenland glacial ice and pan-Arctic sea ice. *Nat. Commun.* **9**, 1038 (2018).

10. Darby, D. A. Ephemeral formation of perennial sea ice in the Arctic Ocean during the middle Eocene. *Nat. Geosci.* **7**, 210-213 (2014).

11. Darby, D. A., Andrews, J. T., Belt, S. T., Jennings, A. E. & Cabedo-Sanz, P. Holocene Cyclic Records of Ice-Rafted Debris and Sea Ice Variations on the East Greenland and Northwest Iceland Margins. *Arctic, Antarctic, and Alpine Research*. **49**, 649-672 (2017).

12. Rubatto, D. Zircon trace element geochemistry: partitioning with garnet and the link between U–Pb ages and metamorphism. *Chem. Geol.* **184**, 123-138 (2002).

13. Andersen, T., van Niekerk, H. & Elburg, M. A. Detrital zircon in an active sedimentary recycling system: Challenging the 'source-to-sink' approach to zircon-based provenance analysis. *Sedimentology*. **69**, 2436-2462 (2022).

14. Puchkov, V. N. & Ivanov, K. S. Tectonics of the Northern Urals and Western Siberia: General History of Development. *Geotectonics*. **54**, 35-53 (2020).

15. Bulatov, T., Yermakov, Y., Kulikova, A., Gareev, B. & Spasennykh, M. Provenance and the U–Pb age constraints on the tuff beds of the Late Jurassic–Early Cretaceous Bazhenovo Formation, West Siberian Basin. *The Depositional Record*. **10**, 312-334 (2024).

16. Akinin, V. V. et al. Episodicity and the dance of late Mesozoic magmatism and deformation along the northern circum-Pacific margin: north-eastern Russia to the Cordillera. *Earth-Sci. Rev.* **208**, 103272 (2020).

17. Campbell, I. H., Czamanske, G. K., Fedorenko, V. A., Hill, R. I. & Stepanov, V. Synchronism of the Siberian Traps and the Permian-Triassic Boundary. *Science*. **258**, 1760-1763 (1992).

18. Parfenov, L. M. et al. Summary of Northeast Asia geodynamics and tectonics. *Stephan Mueller Spec. Publ. Ser.* **4**, 11-33 (2009).

19. Gladkochub, D. P. et al. Petrology, geochronology, and tectonic implications of c. 500 Ma metamorphic and igneous rocks along the northern margin of the Central Asian Orogen (Olkhon terrane, Lake Baikal, Siberia). *J. Geol. Soc.* **165**, 235-246 (2008).

20. Harrison, C. & St-Onge, M. R. Geological history and supercontinent cycles of the Arctic. *Geol. Soc. Am. Bull.* **135**, 162-189 (2022).

21. Rino, S. et al. Major episodic increases of continental crustal growth determined from zircon ages of river sands; implications for mantle overturns in the Early Precambrian. *Phys. Earth Planet. Inter.* **146**, 369-394 (2004).

22. Buffington, J. M. & Montgomery, D. R. A systematic analysis of eight decades of incipient motion studies, with special reference to gravel-bedded rivers. *Water Resour. Res.* **33**, 1993-2029 (1997).

23. García, M. H. Sediment Transport and Morphodynamics. In: Vanoni, V. A., editor. *Sedimentation Engineering*. Reston, VA, USA: American Society of Civil Engineers; 2013. pp. 21-163.

24. Shields, A. Application of similarity principles and turbulence research to bed-load movement: Soil Conservation Service; 1936.

25. Deal, E. et al. Grain shape effects in bed load sediment transport. *Nature*. **613**, 298-302 (2023).

26. Low, H. S. Effect of Sediment Density on Bed-Load Transport. *J. Hydraul. Eng.-Asce*. **115**, 124-138 (1989).

27. Ward, B. D. Relative density effects on incipient bed movement. *Water Resour. Res.* **5**, 1090-1096 (1969).

28. Olhoeft, G. R. & Johnson, G. R. Densities of rocks and minerals. In: Carmichael, R. S., editor. *Practical Handbook of Physical Properties of Rocks and Minerals*. Boca Raton: CRC Press; 1989. pp. 139-175.

29. Dietrich, W. E. Settling velocity of natural particles. *Water Resour. Res.* **18**, 1615-1626 (1982).

30. Shankar, M. S., Pandey, M. & Shukla, A. K. Analysis of Existing Equations for Calculating the Settling Velocity. *Water*. **13**, 1987 (2021).

31. Wu, W. & Wang, S. S. Y. Formulas for Sediment Porosity and Settling Velocity. *J. Hydraul. Eng.-Asce*. **132**, 858-862 (2006).

32. Cheng, N. Simplified Settling Velocity Formula for Sediment Particle. *J. Hydraul. Eng.-Asce*. **123**, 149-152 (1997).

33. Krinsley, D. H. & Doornkamp, J. C. *Atlas of quartz sand surface textures*. (Cambridge University Press, 1973).

34. Mahaney, W. C. *Atlas of sand grain surface textures and applications*. (Oxford University Press, 2002).

35. Stickley, C. E. et al. Evidence for middle Eocene Arctic sea ice from diatoms and ice-rafted debris. *Nature*. **460**, 376-379 (2009).

36. Immonen, N. Surface microtextures of ice-rafted quartz grains revealing glacial ice in the Cenozoic Arctic. *Palaeogeography, Palaeoclimatology, Palaeoecology*. **374**, 293-302 (2013).

37. Whalley, W. B. & Krinsley, D. H. A scanning electron microscope study of surface textures of quartz grains from glacial environments. *Sedimentology*. **21**, 87-105 (1974).

38. Helland, P. E. & Holmes, M. A. Surface textural analysis of quartz sand grains from ODP Site 918 off the southeast coast of Greenland suggests glaciation of southern Greenland at 11 Ma. *Palaeogeography, Palaeoclimatology, Palaeoecology*. **135**, 109-121 (1997).

39. Strand, K., Passchier, S. & Näsi, J. Implications of quartz grain microtextures for onset Eocene/Oligocene glaciation in Prydz Bay, ODP Site 1166, Antarctica. *Palaeogeography, Palaeoclimatology, Palaeoecology*. **198**, 101-111 (2003).

40. Eldrett, J. S., Harding, I. C., Wilson, P. A., Butler, E. & Roberts, A. P. Continental ice in Greenland during the Eocene and Oligocene. *Nature*. **446**, 176-179 (2007).

41. Strand, K. & Immonen, N. Dynamics of the Barents-Kara ice sheet as revealed by quartz sand grain microtextures of the late Pleistocene Arctic Ocean sediments. *Quat. Sci. Rev.* **29**, 3583-3589 (2010).

42. Dunhill, G. Comparison of sea-ice rafted debris; grain size, surface features, and grain shape: U.S. Geological Survey; 1998.

43. St John, K., Passchier, S., TantillO, B., Darby, D. & Kearns, L. Microfeatures of modern sea-ice-rafted sediment and implications for paleo-sea-ice reconstructions. *Ann. Glaciol.* **56**, 83-93 (2015).

44. Mahaney, W. C. Pleistocene and Holocene glacier thicknesses, transport histories and dynamics inferred from SEM microtextures on quartz particles. *Boreas*. **24**, 293-304 (1995).

45. Mahaney, W. C., Claridge, G. & Campbell, I. Microtextures on quartz grains in tills from Antarctica. *Palaeogeography, Palaeoclimatology, Palaeoecology*. **121**, 89-103 (1996).

46. Van Hoesen, J. G. & Orndorff, R. L. A comparative SEM study on the micromorphology of glacial and nonglacial clasts with varying age and lithology. *Can. J. Earth Sci.* **41**, 1123-1139 (2004).

47. Hart, J. K. An investigation of subglacial processes at the microscale from Briksdalsbreen, Norway. *Sedimentology*. **53**, 125-146 (2006).

48. Kalinska-Nartiša, E. et al. Quartz grain features in modern glacial and proglacial environments: A microscopic study from the Russell Glacier, southwest Greenland. *Pol. Polar. Res.* **38**, 265-289 (2017).

49. Rose, K. C. & Hart, J. K. Subglacial comminution in the deforming bed: Inferences from SEM analysis. *Sediment. Geol.* **203**, 87-97 (2008).

50. Woronko, B. Frost weathering versus glacial grinding in the micromorphology of quartz sand grains: Processes and geological implications. *Sediment. Geol.* **335**, 103-119 (2016).

51. Křížek, M., Krbcová, K., Mida, P. & Hanáček, M. Micromorphological changes as an indicator of the transition from glacial to glaciofluvial quartz grains: Evidence from Svalbard. *Sediment. Geol.* **358**, 35-43 (2017).

52. Jacobsen, S. B. & Wasserburg, G. J. Sm-Nd isotopic evolution of chondrites. *Earth Planet. Sci. Lett.* **50**, 139-155 (1980).

53. Niessen, F. et al. Repeated Pleistocene glaciation of the East Siberian continental margin. *Nat. Geosci.* **6**, 842-846 (2013).

54. Batchelor, C. L. et al. The configuration of Northern Hemisphere ice sheets through the Quaternary. *Nat. Commun.* **10**, 3713 (2019).

55. Spielhagen, R. F., Erlenkeuser, H. & Siegert, C. History of freshwater runoff across the Laptev Sea (Arctic) during the last deglaciation. *Glob. Planet. Change*. **48**, 187-207 (2005).

56. Li, Q. et al. Sedimentary record of sea ice rafting in the Laptev Sea during the Holocene: Evidence from the improved ice rafting debris (IRD) proxy. *Palaeogeography, Palaeoclimatology, Palaeoecology*. **660**, 112667 (2025).

57. Dong, J. et al. Enhanced Arctic sea ice melting controlled by larger heat discharge of mid-Holocene rivers. *Nat. Commun.* **13**, 5368 (2022)

58. O'Regan, M. et al. The De Long Trough: a newly discovered glacial trough on the East Siberian continental margin. *Clim. Past*. **13**, 1269-1284 (2017).

59. Polyak, L., Edwards, M. H., Coakley, B. J. & Jakobsson, M. Ice shelves in the Pleistocene Arctic Ocean inferred from glaciogenic deep-sea bedforms. *Nature*. **410**, 453-457 (2001).

60. Jakobsson, M. et al. Evidence for an ice shelf covering the central Arctic Ocean during the penultimate glaciation. *Nat. Commun.* **7**, 10365 (2016).

61. Shen, Z., Yang, C., Zhang, T. & Xu, Y. A more complete and detailed glacial history of the northwestern Chukchi margin—Implications for the existence and evolution of the East Siberian-Chukchi ice sheet. *Quat. Sci. Rev.* **342**, 108915 (2024).

62. Lehmann, C. & Jokat, W. Seismic constraints for ice sheets along the northern margin of Beringia. *Glob. Planet. Change*. **215**, 103885 (2022).

63. Dove, D., Polyak, L. & Coakley, B. Widespread, multi-source glacial erosion on the Chukchi margin, Arctic Ocean. *Quat. Sci. Rev.* **92**, 112-122 (2014).

64. Kim, S. et al. Seismostratigraphic and Geomorphic Evidence for the Glacial History of the Northwestern Chukchi Margin, Arctic Ocean. *Journal of Geophysical Research: Earth Surface*. **126**, e2020JF006030 (2021).

65. Wang, C. Y., Campbell, I. H., Stepanov, A. S., Allen, C. M. & Burtsev, I. N. Growth rate of the preserved continental crust: II. Constraints from Hf and O isotopes in detrital zircons from Greater Russian Rivers. *Geochim. Cosmochim. Acta*. **75**, 1308-1345 (2011).

66. Safonova, I., Maruyama, S., Hirata, T., Kon, Y. & Rino, S. LA ICP MS U–Pb ages of detrital zircons from Russia largest rivers: Implications for major granitoid events in Eurasia and global episodes of supercontinent formation. *J. Geodyn.* **50**, 134-153 (2010).

67. Malkowski, M. A. et al. Continental shelves as detrital mixers: U–Pb and Lu–Hf detrital zircon provenance of the Pleistocene–Holocene Bering Sea and its margins. *The Depositional Record*. **8**, 1008-1030 (2022).
