# Supplementary material for: Arctic zircon U-Pb ages reveal multiphase glaciations in East Siberia during the late Quaternary
Source: Nat Commun. 2025 Aug 13;16:7511. doi: 10.1038/s41467-025-62499-y (PMC12350766; doi:10.1038/s41467-025-62499-y)
Supplement: Supplementary file 2 — Description of Additional Supplementary Information [file 41467_2025_62499_MOESM2_ESM.pdf]

Description of Additional Supplementary Files

File Name: Supplementary Data S1
Description: Water content of cores LV90-8-1 and LV90-9- 1.

File Name: Supplementary Data S2
Description: Detrital zircon U-Pb ages from 17 surface samples from the Arctic continental shelves and 10 core samples from LV90-8-1 and LV90-9-1, including grain size of each zircon grain.

File Name: Supplementary Data S3
Description: Grain size data from cores LV90-8-1 and LV90-9-1.

File Name: Supplementary Data S4
Description: Original records of quartz surface microfeature counting in samples from LV90-8-1 and LV90-9-1.
